# Olive Tree-Ring Problematic Dating: A Comparative Analysis on Santorini (Greece)

**DOI:** 10.1371/journal.pone.0054730

**Published:** 2013-01-28

**Authors:** Paolo Cherubini, Turi Humbel, Hans Beeckman, Holger Gärtner, David Mannes, Charlotte Pearson, Werner Schoch, Roberto Tognetti, Simcha Lev-Yadun

**Affiliations:** 1 WSL Swiss Federal Institute for Forest, Snow and Landscape Research, Birmensdorf, Switzerland; 2 Department of Geography, University of Zurich, Zürich, Switzerland; 3 Laboratory for Wood Biology and Xylarium, Royal Museum for Central Africa, Tervuren, Belgium; 4 PSI Paul Scherrer Institute, Villigen, Switzerland; 5 The Malcolm and Carolyn Wiener Laboratory for Aegean and Near Eastern Dendrochronology, Cornell University, Ithaca, New York, United States of America; 6 Laboratory of Tree-Ring Research, Bryant Bannister Tree Ring Building, University of Arizona, Tucson, Arizona, United States of America; 7 Department of Biosciences and Territory, University of Molise, Pesche, Italy; 8 Department of Biology and Environment, Faculty of Natural Sciences, University of Haifa, Oranim, Tivon, Israel; University of Oxford, United Kingdom

## Abstract

Olive trees are a classic component of Mediterranean environments and some of them are known historically to be very old. In order to evaluate the possibility to use olive tree-rings for dendrochronology, we examined by various methods the reliability of olive tree-rings identification. Dendrochronological analyses of olive trees growing on the Aegean island Santorini (Greece) show that the determination of the number of tree-rings is impossible because of intra-annual wood density fluctuations, variability in tree-ring boundary structure, and restriction of its cambial activity to shifting sectors of the circumference, causing the tree-ring sequences along radii of the same cross section to differ.

## Introduction

Olive trees are a classic component of Mediterranean environments and some of them are known historically to be very old. Olive is one of the first domesticated fruit trees [*1*] and its remnants are found in many archaeological excavations [*2*], [*3*]. The possibility of using olive-wood items for archaeological dating as well as for aging old trees around the Mediterranean and for studying ecological-agricultural-climatic issues is of great interest for many. However, there has not been an attempt to evaluate the possibility of using olive tree-rings for dating.

Every year, at temperate latitudes, trees and shrubs form a new growth-ring. Tree-ring studies, i.e., dendrochronology, enabled scientists to reconstruct past climatic conditions and to date archaeological remains with an annual precision [*4*], [*5*] as well as to calibrate the ^14^C curve for the entire Holocene [*6*]. In tropical regions, annual growth-rings are not systematically formed and many species form non-annual growth-rings or no growth-rings at all [*7*]. In some parts of the Mediterranean region, many woody species are evergreen, and their tree-rings are commonly irregular and not formed annually [*8*].

Trees in the Mediterranean and especially in its eastern part have adapted to the regional climatic conditions characterised by moist cool winters and dry hot summers [*9*]. Given the huge spatio-temporal variability of typical Mediterranean climatic conditions with their common alternating wet and dry spells within the season of cambial activity, with no clear and regular seasonality such as in the temperate region, wood formation is neither always associated with regular dormancy periods nor with the production of definite annual growth-rings [*10*], [*11*]. This is particularly true on small islands, which do not have a pronounced clear seasonality due to the influence of the surrounding large water mass, as was recently shown for trees growing in the island of Elba [*12*], [*13*]. One major problem with dating by tree-rings in Mediterranean environments is that trees do not always form anatomically distinct annual growth-rings, and frequently produce additional intra-annual density fluctuations (IADFs) during drought or temperature fluctuation periods [*8*]. IADFs are morphologically similar to latewood and often it is not easy to distinguish them from boundaries of annual rings, and therefore they are often referred to as “false rings”. They are common in olive wood because the trees are evergreen, and able to restart wood formation after a stress-induced radial growth arrest as soon as environmental conditions allow [*8*]. The olive tree was domesticated and cultivated in the Mediterranean area after the transformation and shaping of the natural ecosystems into managed agro-ecosystems [*1*], where manual weeding, irrigation, training and pruning may induce continuous growth and more IADFs.

Here we describe the results of a blind test conducted involving five tree-ring laboratories to date growth-rings from olive trees currently growing on Santorini, and also to document their wood anatomy, density, and elemental composition, to evaluate whether the number of growth-rings can be counted precisely.

## Materials and Methods

We tested the level of uncertain identification of growth-rings in olive trees growing on Santorini. Cross sections of stems and branches of 37 live trees were sampled in June 2008. No specific permits were required for the described field studies because the trees were growing in abandoned fields, they were not privately owned or protected. All samples were analyzed using standard dendrochronological methods [*14*]. Because the cross-dating was very difficult, we also prepared wood microsections with a sliding microtome, stained them with Safranin and Astra Blue to be analyzed under an Olympus BX41 microscope, using standard wood-anatomical techniques.

Five samples (L8, AT, E2, E3, T) were selected for a blind test involving six dendrochronologists working at the same laboratory (Swiss Federal Research Institute WSL) and four external experts based at: the Laboratory for Wood Biology and Xylarium at the Royal Museum for Central Africa, Tervuren, Belgium; the Wood Anatomy Laboratory, University of Haifa, Oranim, Israel; and two anonymous laboratories (one European and one North American) to count growth-rings.

We also analyzed wood density by Neutron-Imaging Radiography at the cold-neutron-line (ICON) at Paul Scherrer Institute, Villigen, Switzerland [*15*], [*16*]. Scanning X-ray Fluorescence Microscopy (SXFM) using the F3 bending-magnet beamline at the Cornell High Energy Synchrotron Source (CHESS), Cornell University, Ithaca NY, U.S.A., was used to produce elemental maps along measurement radii of four of the samples, to detect elemental boundaries which might help to elucidate true growth-rings from IADFs and bands of phenolic discoloration. Of particular interest are changes in Calcium (Ca) already used to elucidate annual growth in ringless tropical species [*17*], or the impact of precipitation in African *Acacia* spp. [*18*]. Calcium is one of the most abundant and least mobile trace elements analyzed in trees [*19*] and is primarily bound to the cell walls to provide structure and rigidity [*20*], [*21*].

## Results and Discussion

The wood-anatomical structure of *O*. *europea* is characterized by a diffuse porous vessel member arrangement resulting in rather indistinct growth-ring boundaries [*2*]. Vessel members in olive wood are mostly isolated or sometimes arranged in radial files of two to five vessel members. Ray width is commonly biseriate, sometime also uniseriate [*3*]. Measuring growth-rings in olive trees is a complicated task as it is often difficult to distinguish true (annual) growth-rings from IADFs and this is further complicated by the naturally occurring very asymmetric cambial activity [*2*], a phenomenon that becomes more pronounced as olive trees mature. Moreover, in olive wood “pseudo growth-rings” characterize the heartwood as the outcome of patterns of dark phenolic pigmentation that in many cases cross growth-ring boundaries and further complicate the study of wood formation in olive trees.

For the blind test, thin polished stem discs from five olive trunks were sent to all involved laboratories with the request of dating while marking the putative growth-rings with a pencil and giving age estimations for each sample. The WSL laboratory measured these dated radii and compared these data. The average number of counted growth-rings per person shows maximal deviations from the median over all experts from 24.5% (sample AT), to 41.2% (sample E2), 41.2% (sample E3), 50% (sample L8) and 56.3% (sample T). Specific radii in two of the five samples (sample L8: 50% and sample T: 56.3%) reached over 50% deviation from the median.

The implemented blind test contains various sources for uncertainties: imprecision by marking the growth-ring borders, accidental interpretation by the growth-ring width measurement, and uncertainties because of the use of different stem discs although they were thin and followed each other at a distance of only a few millimetres within the stem sample. The analysis may result in different counts of growth-rings, if each radius is analysed independently. Inconsistent counts of growth-rings along one to four radii of each single olive stem disc were made by the different dendrochronologists (Table 1), so there was no agreed growth-ring count. The various irregular patterns of dark discoloration further complicated the growth-ring counts. The number of growth-rings counted on the microsections, with the aid of larger microscope magnifications that eliminated the effect of discoloration, did not match with those of the polished cross sections studied under a binocular microscope. Furthermore, even in individual microsections various anatomical types of putative growth-ring boundaries could be found. Because of all these types of growth-ring structure variability, cross-dating the growth-ring-width series of the samples was impossible.

**Table 1:** Blind test: Counts of growth-rings on the five samples (L8, AT, E2, E3 and T) per expert.

Samples	L8	AT	E2	E3	T
Expert 1 int	9, 10	13, 14	20	24	21
Expert 2 int	9, 9	13, 13	19	15, 16	18, 19
Expert 3 int	7, 7	10, 10, 10	9, 13	14, 15	7, 7
Expert 4 int	7, 8	12, 15	17	19, 24	16
Expert 5 int	8	13, 13	10	17	16
Expert 6 int	7, 9	9, 11	8, 16	13, 13, 16	N
Expert 7 ext	12	14	19	18	20
Expert 8 ext	9, 9	12, 12	14, 14	20, 20	12, 12
Expert 9 ext	7, 8, 8	14, 14	N	N	N
Expert 10 ext	7, 9	14	17, 17	16, 18	11, 21

Experts 1–6 are internal from the Swiss Federal Research Institute WSL, experts 7–10 from four external laboratories: the Laboratory for Wood Biology and Xylarium at the Royal Museum for Central Africa, Tervuren, Belgium; the Wood Anatomy Laboratory, University of Haifa, Oranim, Israel; and two anonymous laboratories (one European and one North American). Some of the experts counted along one radius, some of them along two or three radii of the wood samples. N means that no age determination was possible.

Neutron imaging of the growth-rings shows a similar spectrum of results as the traditional dendrochronological methods. The analyzed samples (L8, AT, E3) could not be dated and dating problems in the samples L8 and AT by neutron imaging occurred at the same locations as in the visual analysis of the stem discs and microsections. The high quality of the neutron image of sample E3 demonstrates the same problems of growth-ring boundary identification. Thus, demonstrating that the problem of dating tree rings is of a biological nature and not a detection problem. However, if the wood fiber direction is not exactly parallel to the direction of the exposure (samples E2, T) no usable results could be achieved. Unfortunately, fiber direction in olive wood changes frequently on a very small scale.

**Figure 1:**
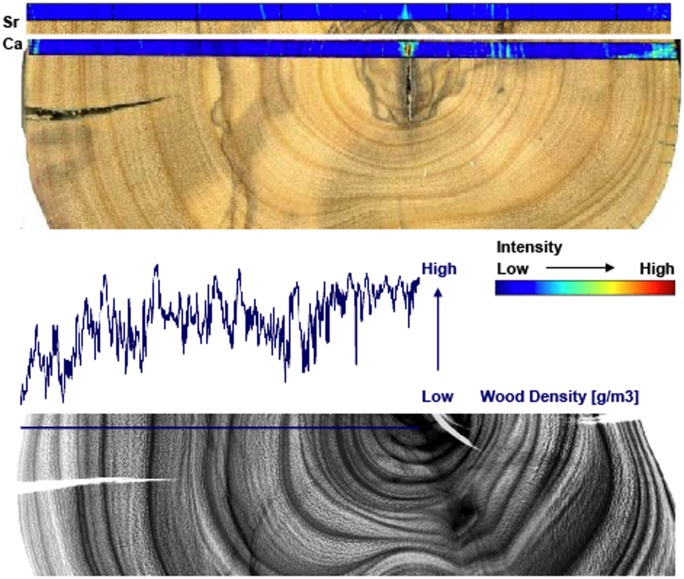
Above: Stem disc from sample E3 overlayed by a SXFM-profile. Content of calcium and strontium increases at the tree-ring borders. Below: Neutron Image of a section of the same sample (E3). Higher density peaks should reflect tree-ring borders but can also be induced by Intra-Annual-Density-Fluctuations, making tree-ring dating impossible.

Neutron imaging improved the definition of wood-density variability in our olive wood samples from Santorini, but also did not enable us reliable identification of annual growth-ring borders (Figure 1). Moreover, the difference between putative annual growth-ring borders and IADFs are not detectable even with Neutron-Imaging Radiography. Our results show that adding high-technology growth-ring identification methods such as Neutron-Imaging Radiography to traditional optical microscopy does not provide better information on the nature of olive tree growth-rings, i.e., allowing to reproducibly determine the number of rings and whether or not they are annual. The problem encountered when dating olive growth-rings is thus not only their basic problematic anatomical nature, but also the inability to distinguish between annual growth-rings versus IADF’s.

**Figure 2:**
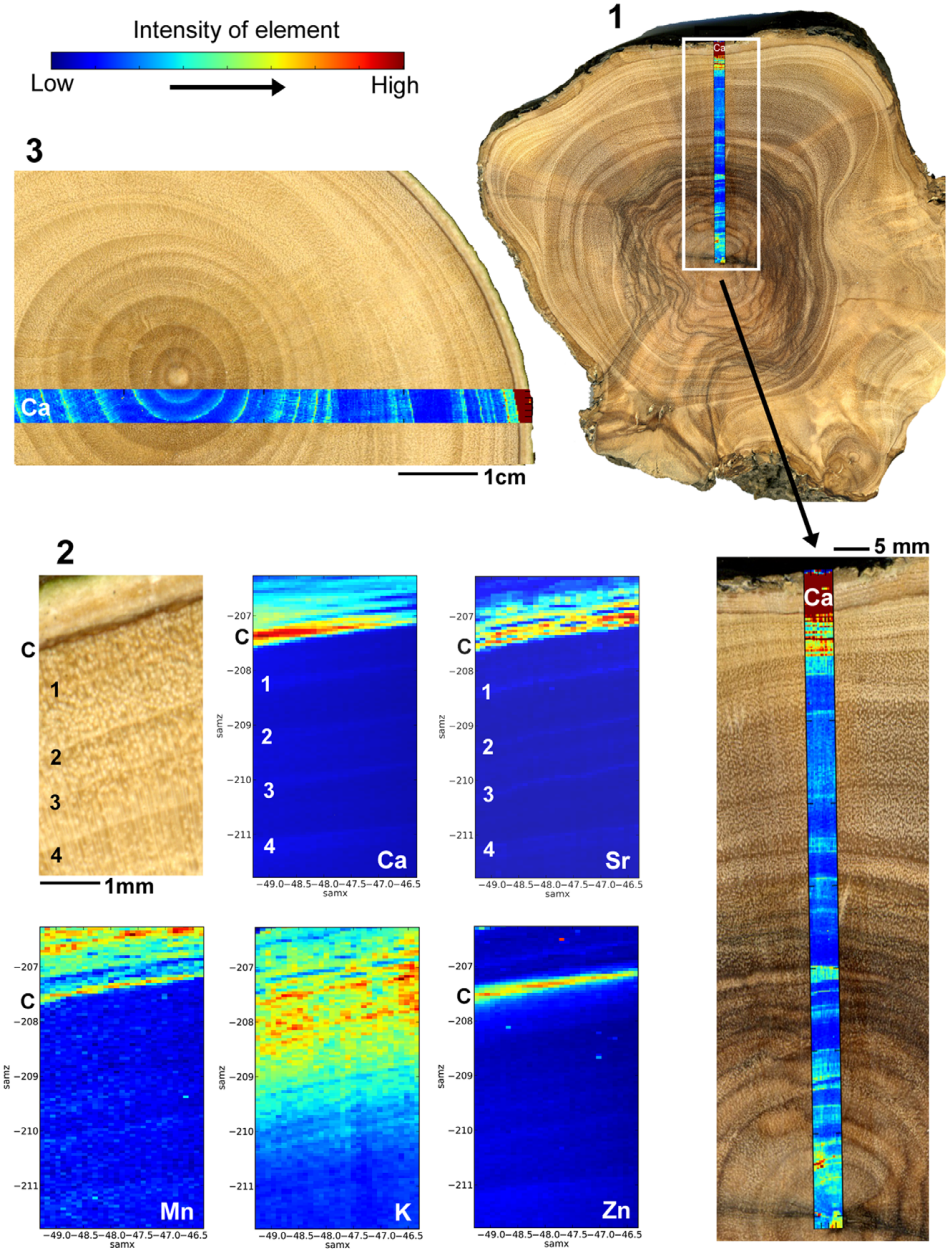
1: Ca intensity mapping can be used to elucidate growth boundaries in areas complicated by phenolic staining. 2: Wood sample (AT) at the cambium/bark interface, and high resolution elemental maps for Ca, Strontium (Sr), Manganese (Mg), Potassium (K) and Zinc (Zn), which show an increase at the cambium ‘C’. Calcium, Sr and Mn also increase in the bark and K increases significantly in the outermost growth rings. Ring boundaries are labelled 1–4 and are marked by an increase in Ca, Sr and to a lesser extent Zn intensity values. IADFs can be observed faintly in the Zn data (where intensity values are very similar for IADFs and ‘true’ boundaries) and to a lesser extent in the Ca data (where a greater contrast in intensity is shown between the two). These observations hold some potential for differentiation between boundaries and IADFs, but the increase in Ca at the boundaries correlates with what can be determined by eye. 3: A lower resolution scan across the opposite radii of the sample AT. Calcium intensity increases match visually determinable ‘boundaries’ with varying degrees of clarity depending on the physical structure of the wood. The main advantage of the mapping is the association of particular elements with that structure, not in providing an alternate means to resolve and count the years of growth represented.

SXFM mapping of Ca intensity showed potential to improve the detection of growth boundaries (and density fluctuations) against phenolic staining (Figure 2, image 1). However, elemental mapping of a number of samples failed to identify alternate elemental patterns within the xylem which might be used to distinguish true annual growth-rings from inter-annual density fluctuations. While the Ca data (especially when compared with Zn patterns) showed some potential for higher intensity in association with potentially ‘true’ growth-ring boundaries, in general the observable changes replicate what can and what cannot be determined from macro- and microscopic analysis of the sample (Figure 1, image 2 and image 3).

The bottom line is that no growth-ring measuring method currently used in dendrochronology, not even the most sophisticated methods, were able to reliably identify the annual growth-ring borders in olive wood from Santorini. The very variable counting results of the blind test by ten well-experienced scientists clearly demonstrates the problem of the identification of olive growth-ring boundaries. There were also large discrepancies in the growth-ring numbers among different radii of the same cross-section, even when analyzed by the same expert, and similar differences among experts. The median value of the growth-rings counted at the WSL on the five samples was, respectively, 8, 13, 14.5, 16.25, and 16, with a maximum difference above and below the median of 44%, whereas at the external laboratories it was 8.5, 14, 17, 18, and 16 (31%).

The fact that even high-technological methods, such as neutron imaging and SXFM analyses, do not aid the identification of true tree-ring borders from IADFs demonstrates that the problematic dating is not a detection problem and instead related to the intrinsical biological nature of olive tree-rings.
